# Functional Haplotypes in *Interleukin 4* Gene Associated with Periodontitis

**DOI:** 10.1371/journal.pone.0169870

**Published:** 2017-01-23

**Authors:** Giovana Anovazzi, Marcell Costa de Medeiros, Suzane Cristina Pigossi, Livia Sertori Finoti, Marcia Pinto Alves Mayer, Carlos Rossa, Raquel Mantuaneli Scarel-Caminaga

**Affiliations:** 1 Department of Oral Diagnosis and Surgery, School of Dentistry at Araraquara, UNESP- Univ Estadual Paulista, Araraquara, São Paulo, Brazil; 2 Department of Morphology, School of Dentistry at Araraquara, UNESP- Univ Estadual Paulista, Araraquara, São Paulo, Brazil; 3 Department of Microbiology, Institute of Biomedical Sciences, University of São Paulo, São Paulo, Brazil; Medical University of South Carolina, UNITED STATES

## Abstract

Chronic periodontitis (CP) is an infectious inflammatory disease that affects tooth-supporting structures and in which dental plaque bacteria, immune mechanisms and genetic predisposition play important roles. Interleukin 4 (IL-4) is a key anti-inflammatory cytokine with relevant action in imbalances in inflamed periodontal tissue. Individuals carrying the TCI/CCI genotype (S-haplotype) of the *IL-4* gene are 5 times more susceptible to CP, whereas the CTI/TTD genotype (P-haplotype) confers protection against CP. Compared with the S-haplotype, subjects with the P-haplotype produce higher levels of the IL-4 protein after non-surgical periodontal therapy. The present in vitro study aimed to investigate the functionality of *IL-4* haplotypes in immune cells to obtain insight into the influence of these genetic variations in regulating immune responses to CP-associated bacteria. Peripheral blood was collected from 6 subjects carrying each haplotype, and their immune cells were challenged with periodontopathogens to compare responses of the different haplotypes with regard to gene expression, protein secretion and the immunophenotype of T helper responses. We found higher *IL-4* mRNA and protein levels in the P-haplotype, which also presented higher levels of anti-inflammatory cytokines. In contrast, cells from S-haplotype subjects responded with higher levels of pro-inflammatory cytokines. S-haplotype individuals exhibited significantly greater polarization toward the Th1 phenotype, whereas the P-haplotype was associated with an attenuated response to periodontopathogens, with suggestive skewing toward Th2/M2 phenotypes. In conclusion, *IL-4* genetic variations associated with susceptibility to or protection against chronic periodontitis are directly associated with influencing the response of immune cells to periodontopathogens.

## Introduction

Chronic periodontitis (CP) is an inflammatory disease that results from interaction between dental biofilm agents and the host immune response [1]. Periodontopathogens, such as *Porphyromonas gingivalis (Pg)*, *Tannerella forsythia*, *Treponema denticola* and *Aggregatibacter actinomycetemcomitans (Aa)*, are abundant in subgingival biofilms at periodontitis-affected sites [2] and trigger an innate host response through Toll-like receptors (TLRs) expressed by resident cells and leukocytes in the periodontal microenvironment [3]. TLR activation induces the production of inflammatory mediators [4–6], which are ultimately responsible for establishing a local immune response. In CP under the continued presence of periodontopathogens, this response consists predominantly of lymphocytes (T-cells) and macrophages [7].

The expression profiles of host response genes influenced by intrinsic genetic variations are important determinants for periodontitis susceptibility [8, 9]. In support of this rationale, differences in levels of interleukins are attributed to polymorphisms in the corresponding genes [10], and genetic susceptibility to periodontal disease has been demonstrated to be associated with variations in genes with functional relevance to the immune response [11, 12].

We previously identified a haplotype formed by -590 (T/C; promoter region, rs2243250), +33 (C/T; 5’UTR region, rs2070874) and VNTR (variable number of tandem repeats; insertion/deletion of 70 bp in intron 3) polymorphisms of the *IL-4* gene that conferred susceptibility to or protection against the development of CP. Individuals carrying the genotype TCI/CCI (S-haplotype) were found to be five times more susceptible to CP (OR_adjusted_ = 5.27, 95% CI = 2.28–12.18), whereas those carrying genotype CTI/TTD (P-haplotype) appeared to be genetically protected from developing periodontitis (OR_adjusted_ = 0.29, 95% CI = 0.08–0.88) [13]. Subsequent clinical studies demonstrated higher levels of IL-4 with the P-haplotype after 45 days of non-surgical periodontal therapy [14]; the S-haplotype, which was previously associated with genetic susceptibility to CP, was associated with increased levels of periodontopathogenic bacteria [15].

Based on these findings from previous case-control and clinical studies, we sought to assess the functionality of these *IL-4* haplotypes in the response of immune cells to periodontopathic bacteria. In this study, we examine the impact of these *IL-4* haplotypes on gene expression regulation, cytokine production and T helper-type polarization of peripheral blood cells in response to periodontopathogens.

## Materials and Methods

### Subjects

The TCI/CCI (S-haplotype) or CTI/TTD (P-haplotype) genotype of the *IL-4* gene in individuals was confirmed by sequencing. Sample size calculation was performed with DDS Research (Sample Size Calculator, Average, two sample) utilizing *IL-4* gene expression values for samples from a pilot study. This calculation determined six subjects of each genotype, with the ability to detect as significant (at the 80% confidence level) differences of 0.3 units in the averaged values with an estimated variation of 0.6 units. Utilizing the obtained data at the end of this study, power calculation analyses were also performed by DDS Research, showing 100% statistical power for each evaluation, i.e., gene expression, protein concentration and flow cytometry. Therefore, a total sample size of 6 individuals provided sufficient power for detecting differences between the groups regarding the evaluated data.

Periodontal clinical examinations were performed on all subjects, who were considered periodontally healthy (exhibited no gingival bleeding and PPD ≤ 3 mm). Exclusion criteria were as follows: history of subgingival periodontal debridement or periodontal surgery in the preceding 6 months; use of antibiotics, anti-inflammatory or corticoids in the four last months; smoking; history of systemic or local disease with influence on the immune system; current pregnancy or lactation.

### Ethics statement

This study was approved by the Human Research Ethics Committee of School of Dentistry at Araraquara, UNESP, Brazil (Certificate of Presentation to Ethics Assessment -CAAE 18527813.7.0000.5416). The subjects provided written informed consent to participate in this study.

### Bacterial growth conditions and stimulation

A frozen stock of *Porphyromonas gingivalis (Pg)* strain ATCC 33277 was cultured on plates of Tryptic Soy Agar supplemented with 5% defibrinated sheep blood, 0.5 mg/mL hemin and 1 mg/mL menadione. A frozen stock of *Aggregatibacter actinomycetemcomitans (Aa)* strain JP2 was culture on Tryptic Soy Agar containing 0.6% w/v yeast extract. Both cultures were maintained in an anaerobic chamber at 37°C in 85% N_2_, 5% CO_2_ and 10% H_2_ for 2 to 3 days and harvested at the mid-logarithmic phase of growth. To calculate the amount of bacteria for the stimulus we adjusted an OD of 0.5 (*Pg*) and 0.2 (*Aa*) in a wavelength of 495 nm which was determined to correlate to 10^7^ CFU/ml. For the gene expression and cytokine production assays, alive bacteria were used in the proportion of 100:1 (bacteria:cells). For the flow cytometry experiments, bacteria were heat-inactivated, at 65°C for 1 hour in a water-bath in the proportion of 100:1 (bacteria:cells).

### Gene expression

Peripheral blood from subjects was drawn into a vacutainer blood-collecting tube (Becton, Dickinson and Company) with EDTA/K3. Separation of nucleated cells was performed using a double gradient of Histopaque (Sigma Chemical). After initial separation of neutrophils and PBMCs, monocytes and lymphocytes were isolated from other PBMCs using negative selection magnetic bead-based sorting (Dynabeads untouched human monocytes kit; Dynabeads untouched human T cells kit, Invitrogen). After separation, all cells were cultured overnight in RPMI 1640 supplemented with 1% heat-inactivated FBS. Lymphocytes were activated with anti-CD3/CD28 antibodies (Dynabeads Human T-Activator, Life Technologies) for 7 hours. After activation, the cell concentration was adjusted to 5x10^5^ cells/mL for 4-hour stimulation (period established from the pilot study) with *Pg or Aa* resuspended in medium or Control (same volume of medium used to resuspend bacteria). Total RNA was extracted using an affinity column system that included treatment to eliminate possible genomic DNA contaminants (RNAqueous kit, Ambion Inc.). The RNA was quantitated using a microvolume spectrophotometer (NanoView, GE Healthcare), and 300 ng was used for cDNA synthesis with random hexamer primers and reverse transcriptase (High-Capacity cDNA Reverse Transcription kit, Applied Biosystems). Real-time PCR was performed using TaqMan chemistry (TaqMan Fast Advanced Master Mix) and pre-designed and optimized sets of primers and probe (Gene expression assays, Applied Biosystems) for detecting *IL-4* (Hs00174122_m1*)*, *IL-8* (Hs00174103_m1), *IL-12* (Hs01073447_m1), and *TNFA* (Hs01113624_g1). Expression of *GAPDH* (Hs02758991_g1) was used as the endogenous control. The data were analyzed as relative changes to unstimulated controls using the ΔΔCt method with the thermocycler’s software (StepOne Plus, Applied Biosystems).

### Cytokine production

Equal volumes of whole blood containing 1x10^6^ PBMCs and RPMI 1640 supplemented with 20% heat-inactivated FBS were combined and immediately stimulated with *Pg* and *Aa* for 12 h at 37°C in a 5% CO_2_ atmosphere. The levels of selected candidate proteins, GM-CSF (granulocyte-macrophage colony-stimulating factor), TNF-α, IL-1β, IL-4, IL-6, MIP-1β (macrophage inflammatory protein-1β), eotaxin, RANTES (regulated on activation, normal T cell expressed and secreted), MIG (monokine induced by gamma interferon), IL-12 (p40/p70), IL-8, IL-17, MIP-1α (macrophage inflammatory protein 1 alpha), IL-10, IL-1RA, IFN-γ, IL-13, MCP-1, IL-7, IL-15, IFN-α, IL-2R, IP-10, IL-5, and IL-2, in whole blood were measured using a bead-based multiplex assay (Human Cytokine 25-Plex Panel, cat#LHC0009, Invitrogen) with a Bio-plex 200 (Bio-Rad) following the manufacturer’s instructions.

### Immunophenotyping

Equal volumes of whole blood containing 1x10^6^ PBMCs and RPMI 1640 supplemented with 20% heat-inactivated FBS were combined and immediately stimulated with *Pg* and *Aa* for 72 h at 37°C in a 5% CO_2_ atmosphere. Non-attached cells were collected by centrifugation (400xg, 5 min, RT), counted and adjusted to 1x10^6^ cells/mL. These samples were then separated into three aliquots: two aliquots were stained for CD4/PeCy7 (T helper cell marker; BD Biosciences) and the remaining aliquot for CD14/PeCy7 (monocyte marker; BD Biosciences) for 30 minutes in the dark. Cells in both aliquots were permeabilized with saponin-containing buffer (Cytoperm, BD Biosciences) for 15 minutes. The two CD4-stained aliquots were subsequently stained for IFN-γ-FITC/IL-4 PE or for IL-17 PE/FoxP3-AlexaFluor488; the CD14-stained aliquots were stained for IL-12 FITC and IL-10 PE. Staining was performed for 40 minutes in the dark according to the manufacturer’s instructions. Both unstained samples and isotype-control samples were prepared using the same protocol. Data were acquired using a flow cytometer (FACSVerse, BD Biosciences) at 488 nm laser excitation and analyzed using the cytometer’s software (BD FACSuite, BD Biosciences).

### Statistical analysis

To compare the outcomes of interest according to *IL-4* haplotype, the normality of the data was analyzed using the D’Agostino-Pearson test; then non-parametric tests were performed (Mann-Whitney) utilizing GraphPad Prism software (GraphPad Software, Inc.). Differences were considered statistically significant at p < 0.05. For all outcomes three independent experiments were performed in triplicate for each stimulus.

## Results

No differences between the groups regarding the demographic profile and periodontal status of the subjects (Table 1) were observed (p > 0.05).

**Table 1:** Demographic profile and periodontal variables of the patients enrolled in the study.

Haplotypes/Characteristics	CTI/TTD (N = 6)	TCI/CCI (N = 6)
**Median Age** (min-max)	40 (36–45)	39 (37–46)
**Gender** n (%)		
Female	4 (90.0)	4 (90.0)
Male	2 (10.0)	2 (10.0)
**N° of teeth** (median (min-max))	28 (25–29)	27 (25–28)
**BoP** (%; median (min-max))	1.20 (0.99–1.31)	1.27 (0.97–1.41)
**PPD** (mm; median (min-max))	1.26 (1.04–1.58)	1.76 (1.21–2.25)
**CAL** (mm; median (min-max))	1.44(1.05–2.29)	1.76(1.21–2.31)

N = individuals number; % = percentage of the number of individuals; BOP = Bleeding on Probing; PPD = Periodontal Probing Depth; CAL = Clinical Attachment Level

### Influence of *IL-4* haplotypes on cytokine gene expression by different immune cells

**Fig 1:**
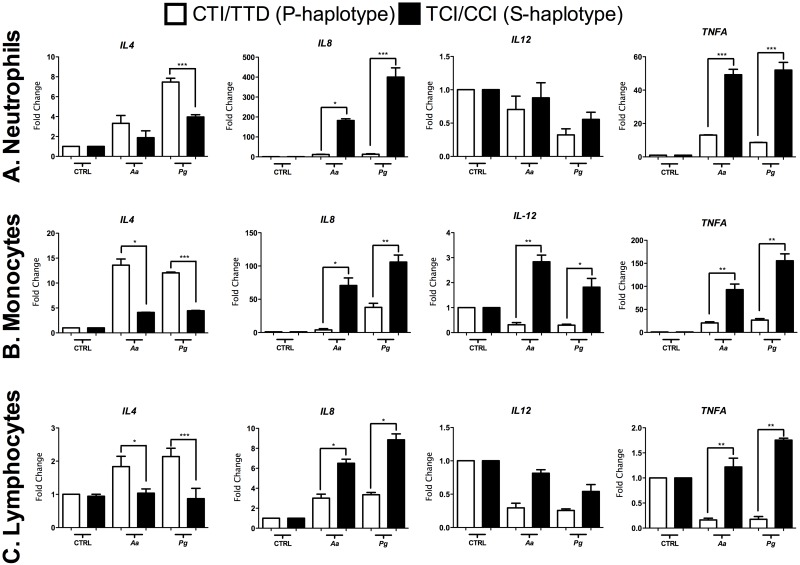
Gene expression (mRNA) levels of IL-4, IL-8, IL-12 and TNFA in isolated cells from the peripheral blood of subjects carrying susceptibility or protection haplotypes after *Aa* and *Pg* stimulation. **A.** Neutrophils, **B.** monocytes, **C.** lymphocytes. The results are represented as the fold change (normalization to the endogenous control). *p < 0.05; ** p < 0.01; *** p < 0.001. The Mann-Whitney test.

The expression levels of *IL-4*, *IL-8*, *IL-12* and *TNFA* by neutrophils, monocytes and lymphocytes are shown in Fig 1. Neutrophils (Fig 1A), monocytes (Fig 1B) and lymphocytes (Fig 1C) from P-haplotype individuals expressed significantly higher levels of *IL-4*, and *Aa*, *Pg* induced higher levels of *IL-4* in neutrophils and lymphocytes.

*IL-12* gene expression by neutrophils and lymphocytes was reduced after bacterial stimulation, regardless of haplotype (Fig 1A and 1C). Interestingly, IL-12 expression by monocytes from S-haplotype subjects was significantly greater than that by monocytes from P-haplotype subjects (Fig 1B).

Among the selected candidate cytokine genes expressed by neutrophils and lymphocytes, *IL-8* was the most prominently expressed. Both *Pg* and *Aa* significantly induced *IL-8* mRNA, which was greater in cells from S-haplotype subjects (Fig 1A–1C).

*TNFA* gene expression was also induced by both *Pg* and *Aa*; however, *Pg* was a more potent stimulus overall. Statistically higher levels were observed in neutrophils, monocytes and lymphocytes from S-haplotype individuals (Fig 1).

### Cytokine production in whole blood and *IL-4* haplotypes

**Fig 2:**
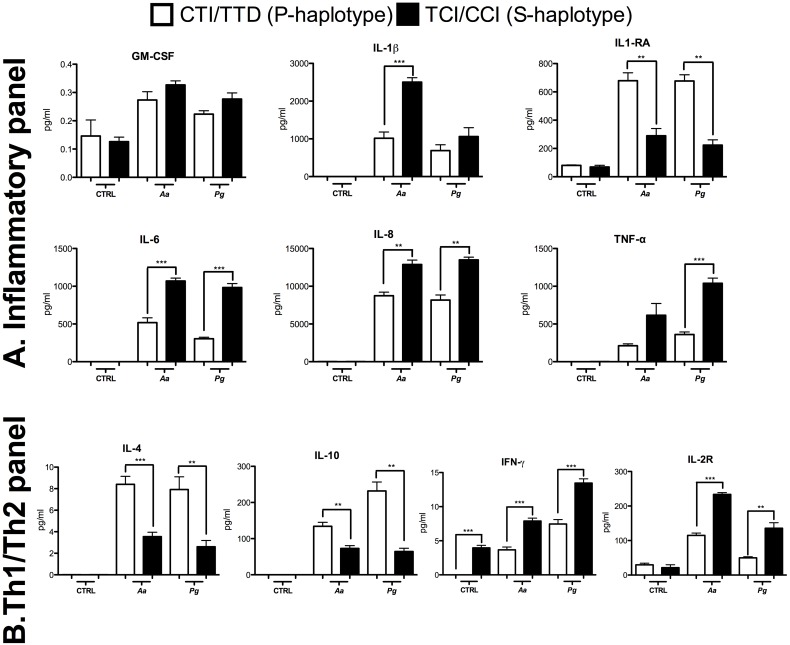
**A.** Inflammatory Panel **B.** Th1/Th2 Panel represented by the proteins analyzed by a multiplex assay in the whole blood of subjects carrying susceptibility or protection haplotypes after *Aa* and *Pg* stimulation. The concentration is expressed in pg/mL. *p < 0.05; ** p < 0.01; *** p < 0.001. The Mann-Whitney test.

Of the 25 cytokines included in the assay, IL-2, IL-5 and IL-17 were not detected. In the S-haplotype, we observed increased levels of IL-1β, IL-6, IL-8 and TNFA pro-inflammatory cytokines. However, significantly higher levels of the IL-1RA receptor were found in P-haplotype cells after bacteria stimulation (Fig 2A).

Production of cytokines associated with the Th1/Th2-type response suggests that stimulation with *Aa* and *Pg* results in a shift toward the Th2 response in the P-haplotype, as represented by increased levels of IL-4 and IL-10. In contrast, the same bacterial stimuli resulted in a possible skewing toward a Th1 response in the S-haplotype, as indicated by significantly higher levels of IFN-γ. Higher production of IL-2R was observed in the S-haplotype after bacterial stimulation (Fig 2B).

**Fig 3:**
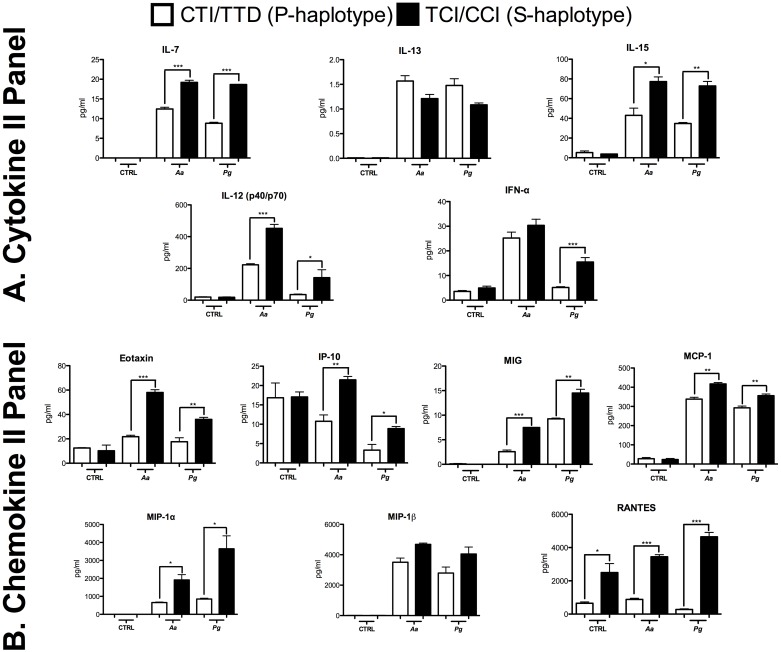
**A.** Cytokine II Panel, **B.** Chemokine II Panel represented by the proteins analyzed by a multiplex assay in the whole blood of subjects carrying susceptibility or protection haplotypes after *Aa* and *Pg* stimulation. The concentration is expressed in pg/mL. *p < 0.05; ** p < 0.01; *** p < 0.001. The Mann-Whitney test.

Based on Cytokine II Panel, we observed significantly higher levels of IL-7, IL-15, and IL-12 in cells from the S-haplotype after stimulation by both bacteria. However, for IFN-α, a significant difference was observed with *Pg*. Although there were higher IL-13 levels in the P-haplotype, the difference was not significant (Fig 3A).

Levels of chemokines Eotaxin, IP-10, MIG, MCP-1, MIP-1α and RANTES were all significantly higher in the S-haplotype in the presence of bacterial stimulation. Notably, *Aa* was more potent than *Pg* only with regard to inducing Eotaxin and IP-10, whereas MIG, MIP-1α and RANTES were more strongly induced by *Pg*. There were no significant differences for MIP-1β production (Fig 3B).

### Polarization of monocytes and T helper cells

**Fig 4:**
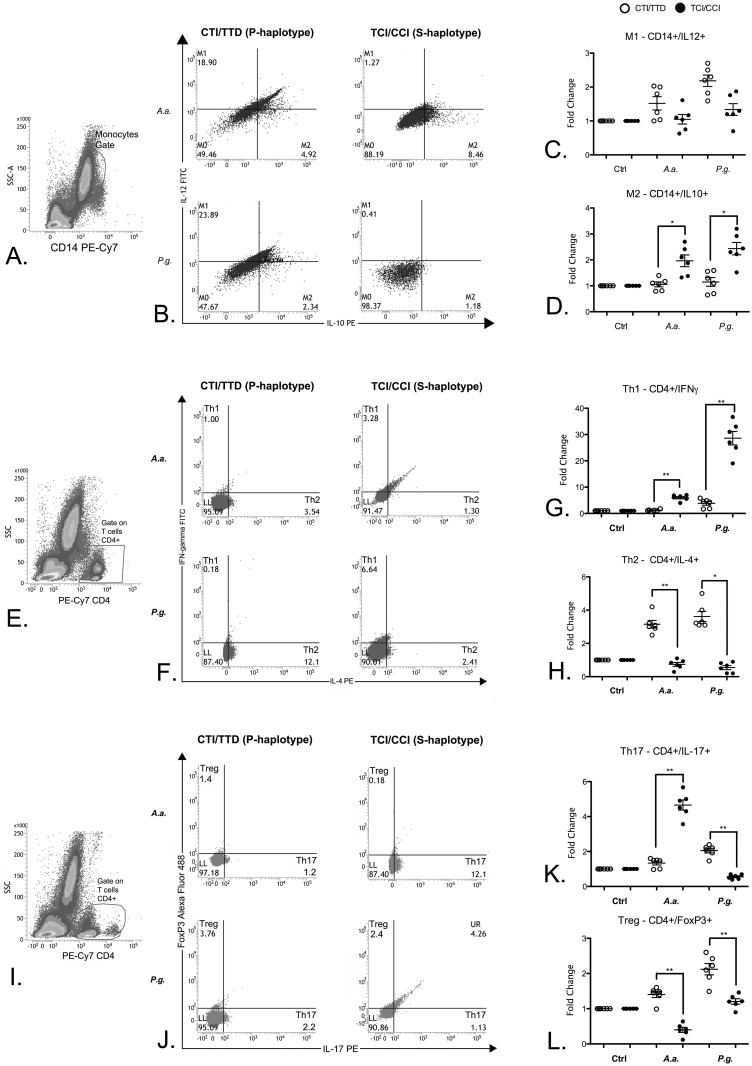
Profiles assessed by flow cytometry in whole from of subjects with susceptibility or protection haplotypes after *Aa* and *Pg* stimulation. **A.** Monocyte gate; **B.** dot-blot representative of M1 and M2; **C.** fold change of all subjects of the M1 profile; **D.** fold change of all subjects of the M2 profile; **E.** CD4+ gate; **F.** dot-blot representative of Th1 and Th2; **G.** fold change of all subjects of the Th1 profile; **H.** fold change of all subjects of the Th2 profile; **I.** CD4+ gate; **J.** dot-blot representative of Th17 and Treg; **K.** fold change of all subjects of the Th17 profile; **L.** fold change of all subjects of the Treg profile. *p < 0.05; ** p < 0.01. The Mann-Whitney test.

Monocytes from subjects with the P-haplotype were more prone to skewing toward an M1 activation profile after stimulation with *Aa* and *Pg*. Despite the greater general response caused by stimulation with *Pg*, there was no significant difference between the haplotypes for the IL-12^high^/IL-10^low^ profile (Fig 4C). Monocytes from S-haplotype subjects showed a significantly higher polarization toward the alternative activation phenotype M2 (IL-12^low^/IL-10^high^) after bacterial stimulation (Fig 4D). In agreement with a ‘pro-inflammatory’ skewing of immune cells from subjects with the S-haplotype, CD4+ cells from these subjects showed significantly higher polarization toward pro-inflammatory Th1 and Th17 phenotypes after stimulation with both *Aa* and *Pg* for Th1 but only with *Aa* stimulation for Th17 (Fig 4G and 4K). Interestingly, stimulation with Pg induced a greater number of Th17 cells in P-haplotype individuals. CD4+ cells from these subjects also presented a significantly greater proportion of Th2 and Treg phenotype cells (Fig 4H and 4L), indicating an overall anti-inflammatory or immunosuppressive response in this haplotype.

## Discussion

Here, we investigate the functionality of haplotypes of the *IL4* gene with regard to the response of immune cells to periodontophatic bacteria. Due to the homogeneity of demographic data of the subjects carrying each haplotype and the lack of difference in periodontal clinical parameters, these characteristics were not variables of influence in this study.

P-haplotype subjects showed higher *IL-4* mRNA expression by bacterially stimulated neutrophils, monocytes and lymphocytes, indicating that these genetic variations are functional and increase the transcriptional activity of the *IL4* gene in immune cells. The levels of IL-4 in whole blood were also significantly higher in cells from P-haplotype individuals after stimulation with both *Aa* and *Pg*, suggesting modulation of the entire immune response. This immunomodulatory effect is supported by the increased proportion of Th2 cells in the P-haplotype after stimulation with *Aa* and *Pg*. In agreement with our findings, the TTD *IL-4* haplotype was previously associated with increased production of IL-4 [16], and T helper cells with the CCI haplotype produce significantly less IL-4 [17]. These findings agree with our observation of a relationship between lower *IL-4* gene and protein expression and the CCI haplotype, even in heterozygosis (TCI/CCI, S-haplotype). Conversely, Bartova et al. [18] did not find significant differences, though they investigated only 2 (-590 C/T and intron 3 VNTR) of the 3 polymorphisms that form the *IL-4* haplotype.

We evaluated whether the *IL-4* gene haplotypes previously associated with CP (Anovazzi et al. 2010) could influence gene expression and protein production of certain key immune mediators as well as the phenotype of T helper cells and monocytes in response to stimulation with *Aa* and *Pg*. Initially, we assessed the response of neutrophils, monocytes and CD4+ lymphocytes as selected immune cells. All cells types carrying S-haplotype presented increased levels of IL-8 and TNF-α (mRNA and protein) after stimulation with both *Aa* and *Pg*. The present study demonstrates for the first time the relationship between *IL-4* gene haplotypes and *IL-8* gene expression in leukocytes after stimulation with periodontopathogens. Because IL-8 mediates neutrophil migration and activation from peripheral blood to tissue [19, 20] and TNFA is a potent pro-inflammatory cytokine that increases the production of collagenases, prostaglandins, chemokines, cell adhesion molecules and bone resorption-related factors [21, 22], increased expression of these mediators by bacterial-stimulated leukocytes from S-haplotype subjects may correlate with more pronounced inflammation and greater severity of periodontitis.

In agreement with the enhanced pro-inflammatory response associated with the S-haplotype, levels of Th1/M1 phenotype-associated IL-12 were significantly higher in monocytes of this haplotype. However, the results for M1/M2 polarization may be influenced by the fact that we used monocytes and not macrophages in these experiments. IL-12 stimulates the production of IFN-γ and TNFA by T cells and natural killer (NK) cells, which suppress production of IL-4 [23]. In agreement with these findings, we observed that CD4+ cells from subjects with the S-haplotype exhibited significantly more pronounced polarization toward the Th1 phenotype (CD4+, IFN-γ+), which may be driven by the increased expression of IL-12 in association with reduced levels of IL-4. Tregs are essential for maintaining peripheral tolerance to downregulate the immune response, whereas Th17 cells play a critical role in several autoimmune diseases, inflammation and host defense [24]. Polarization toward Th17 was significantly higher in the S-haplotype after stimulation with *Aa*; however, upon stimulation with *Pg*, the proportion of Th17 cells was higher in the P-haplotype. This differential regulation of Th17 polarization may be related to the serotype of the bacteria and their associated virulence factors and antigens. *Aa* (b serotype) is known to induce more exacerbated Th17 polarization [25] compared to the *Pg* (ATCC 33277) serotype [26]. Therefore, the S-haplotype exhibited a more pronounced Th17 response to a stronger activator of the phenotype and a reduced Th17 response to a weaker inducer. Another possible explanation is that Th17 is a phenotype characterized by plasticity (may convert into Treg cells) and heterogeneity (there are immunosuppressive FOXP3+/RORc+/IL17+ Th17 cells) [27], which the limitations in our experimental approach did not allow us to detect.

Our experiments performed using whole blood allowed for detecting interaction among diverse immune cells, which is more representative of the effects of *IL-4* haplotypes on the immune response in vivo but is less specific in terms of the effect of the *IL-4* haplotype of each immune cell type. Stimulation with *Aa* and *Pg* resulted in a higher production of IL-10 in the P-haplotype. As IL-10 is a cytokine with anti-inflammatory properties and a central role in limiting the host tissue damage associated with overt immune response [28], an IL-10-attenuated immune response may justify the reduced susceptibility to CP in the P-haplotype. Differences in the levels of IL-10 are explained mainly by the specificity of flow cytometry in detecting intracellular IL-10 in CD14+ cells; when using whole blood, IL-10 secreted by different immune cells, including dendritic cells and B and T lymphocytes, is also detected [29].

In general, the S-haplotype was associated with increased production of pro-inflammatory cytokines and chemokines, including IL-1B, IL-6, IL-7, IL-15, IFN-α, Eotaxin, IP-10, MIG, MCP-1, MIP-1α, RANTES, and IL-2R. In sharp contrast, the P-haplotype was associated with increased levels of anti-inflammatory mediators, such as IL-10, IL-13 and IL-1-RA. Similarly, Bartova et al. [18] verified that *IL-4* polymorphisms (-590 C/T and intron 3 VNTR) increase the production of IL-6, IFN-γ and IL-1β after bacterial stimulation.

In summary, we show that immune cells from subjects presenting the P-haplotype respond with significantly greater expression of IL-4 in response to stimulation with periodontopathogens. This finding supports previous clinical observation of a higher IL-4 concentration (pg/μL) in the gingival crevicular fluid (GCF) of subjects carrying this haplotype after 45 days of non-surgical periodontal therapy compared with the S-haplotype [14]. In addition, by assessing both the response of immune cell types individually and combined, we provide further insight into the influence of these haplotypes on the *IL-4* gene in the immune response. The P-haplotype was associated with an attenuated response to the tested periodontopathogens, with a suggestive skewing toward the Th2/M2 phenotypes. In sharp contrast, cells from subjects carrying the S-haplotype responded to the same bacterial stimuli with significantly greater production of pro-inflammatory mediators and a more pronounced shift toward the Th1 phenotype. These results not only indicate that the specific genetic variations studied are functional in immune cells but also provide biological support for previous clinical findings associating reduced susceptibility to chronic periodontitis with the P-haplotype [13].

It is important to consider that we used an in vitro approach in which only immune cells were assessed, without the participation of important stromal cells involved in the pathogenesis of CP. For practical reasons, we arbitrarily selected only two gram-negative microorganisms associated with periodontal disease: due to their distinct potencies in activating TLR2/TLR4 and also because of the distinct virulence factors expressed by these bacteria (e.g., leucotoxin). Thus, the lack of a complex biofilm and also the intensity of the bacterial stimulation are not commensurate with periodontal disease clinically. Another aspect to be weighted in the interpretation of these results is the limited number of subjects presenting each genotype, as the specific *IL-4* gene haplotypes studied may be accompanied by variability in other regions of the same gene and by a myriad of genetic variations in other genes relevant to the immune response. In spite of these limitations, this study provides consistent and relevant information on the functional role of these *IL-4* gene haplotypes with regard to the response of immune cells to periodontopathogenic bacteria, considering the different immune cells both separately and combined.

In conclusion, we demonstrate the functional role of the investigated *IL-4* haplotypes; the results might biologically explain our findings in previous case-control and clinical studies. Moreover, these haplotypes in the *IL-4* gene have significant effects on the response of immune cells to gram-negative bacteria, which may provide significant information for other conditions involving host-microbial interactions, such as colitis and sepsis.
